# Dataset for the assessment of metallic pollution in the Saint-Charles River sediments (Québec City, QC, Canada)

**DOI:** 10.1016/j.dib.2019.104256

**Published:** 2019-07-15

**Authors:** Léo Chassiot, Pierre Francus, Arnaud De Coninck, Patrick Lajeunesse, Danielle Cloutier, Thibault Labarre

**Affiliations:** a INRS-ETE, 490 rue de la Couronne, Québec, QC, G1R 9A9, Canada; b GEOTOP, Geochemistry and Geodynamics Research Center, Montreal, Canada; c Département de Géographie, Université Laval, Pavillon Abitibi-Price, 2405 Rue de la Terrasse, Québec, QC, G1V 0A6, Canada; d Centre d’Études Nordiques, Université Laval, Pavillon Abitibi-Price, 2405 Rue de la Terrasse, Québec, QC, G1V 0A6, Canada

**Keywords:** Urban river, Reservoir sediments, Pollution, Heavy and trace metals

## Abstract

This Data in Brief article presents sedimentological and geochemical parameters from a set of sedimentary samples collected in the Saint-Charles River, a tributary of the Saint-Lawrence River flowing in Québec City (QC, Canada). It details the experimental design, methods, materials and results of destructive analyses related to a multi-proxy study of polymetallic contamination in sediments collected within an urban reservoir (Spatial and temporal patterns of metallic pollution in Québec City, Canada: Sources and hazard assessment from reservoir sediment records, https://doi.org/10.1016/j.scitotenv.2019.04.021, (Chassiot et al., 2019)). The present article summarizes the results of relevant parameters on a set of 68 samples: total organic carbon (TOC), sulfur content, grain-size, and concentrations of heavy and trace metals. It also presents the calculation of enrichment factors, geoaccumulation indexes, and metallic pollution index.

**Table:** Specifications table

Subject area	*Geochemistry and sedimentology*
More specific subject area	*Organic carbon, grain-size, heavy and trace metals*
Type of data	*Tables and charts*
How data was acquired	*CHNS Analyzer (Truspec), Laser Particle Size Analyzer (Horiba LA-950), ICP-AES Varian X*
Data format	*Raw and analyzed*
Experimental factors	*HCl (CHNS), No pretreatment (grain-size), HNO*_*3*_ + *HCLO*_*4*_ + *HF (ICP-AES)*
Experimental features	*Multi-proxy analysis of sediment samples, including sedimentological analyses and geochemical survey (analyses conducted on a set of 68 samples).*
Data source location	*Québec City (QC, Canada)*
Data accessibility	*Data available in this article*
Related research article	*Chassiot, L., Francus, P., De Coninck, A., Lajeunesse, P., Cloutier, D., Labarre, T., Spatial and temporal patterns of metallic pollution in Québec City, Canada: sources and hazard assessment from reservoir sediment records. Sci of the Tot Environ 673, 2019, 136–147.**[1]*

**Table:** 

**Value of the data** • A geochemical and sedimentological dataset to document metallic pollution and associated environmental hazards in Québec City. • A dataset to be considered for local restoration plans and urban management policies, as well as pollution issues within the Saint-Lawrence Estuary. • A benchmark for future studies dedicated to pollutants in urbanized environments across Canada. • A support for multi-disciplinary research in urban centers and urban reservoirs.

## 1 Data

Data presented in this article are related to a multi-proxy study of pollution in the sediments of the Saint-Charles River, a tributary of the Saint-Lawrence River flowing in Québec City [1]. The present article focuses on destructive analyses used to acquire sedimentological and geochemical data, in complement to non-destructive analyses and age-depth model presented in Chassiot et al. [1]. Sedimentological and geochemical data include total organic carbon (TOC), sulfur (S), grain-size, and heavy and trace metals content for silver (Ag), arsenic (As), cadmium (Cd), cobalt (Co), chromium (Cr), copper (Cu), mercury (Hg), manganese (Mn), molybdenum (Mo), nickel (Ni), tin (Sn), lead (Pb), vanadium (V), and zinc (Zn).

A total of 68 samples is presented. Among them, a first dataset of 39 samples (Table 1) includes 6 surface samples collected at the intersection between the Saint-Charles River and its tributaries (JAU, NEL, LOR, BER, CAR, and LAI), 3 surface samples collected in the downstream section (VER, DRA, and FLE), and 30 samples (A, B, and C) extracted from a series of short-cores collected in the river channel (RSC16-01 to −08, BER16, and FLE17). The second dataset consists in 29 samples extracted from long-core RSC17 (Table 2) to document the historical distribution since the creation of the reservoir in the early 1970s [1].

**Table 1:** Geochemical and sedimentological parameters in surface sediment and short-core samples, including heavy and trace metal content in mg/kg, TOC (%), S (mg/kg), and fine fraction (silts and clays) in %. The reference sample represents the background geochemistry of the studied area [1]. Data are listed following an upstream-downstream transect. A, B, and C refer to top, middle, and bottom-core samples, respectively [1]. Limits of Detection (LOD) include analytical precision and dilution factors. n.a. = not analyzed. Int. Fe = analytical interference with iron.

ID	Ag mg/kg	As mg/kg	Cd mg/kg	Co mg/kg	Cr mg/kg	Cu mg/kg	Mn mg/kg	Mo mg/kg	Ni mg/kg	Pb mg/kg	Sn mg/kg	V mg/kg	Zn mg/kg	Hg ng/g	Ti mg/kg	TOC %	S mg/kg	Silts + clays %
*LOD*	*0.9*	*5*	*0.2*	*2*	*0.5*	*0.8*	*0.3*	*0.8*	*0.6*	*5*	*3*	*0.5*	*1*	*0.03*	*3*	*0.05*	*30*	
REFERENCE	0.04	4.58	0.75	14.08	25.96	9.29	474.55	2.29	13.73	12.75	2.05	68.24	207.66	62.33	4344.02	7.90	1243	55.20
JAU	<0.9	5.32	<0.2	1.87	5.53	<0.8	151.06	<0.08	3.09	16.28	<3	10.32	45.74	1.98	788.30	<0.05	22	0.00
NEL	<0.9	<5	<0.2	1.71	2.32	<0.8	148.99	<0.08	1.41	15.81	<3	8.46	42.28	2.94	943.29	0.13	29	0.00
LOR	<0.9	<5	<0.2	2.54	10.78	8.00	188.89	<0.08	5.89	10.00	<3	16.11	34.44	5.31	1144.44	0.28	400	0.40
BER	<0.9	<5	0.12	2.32	16.89	2.90	240.76	<0.08	8.70	15.50	<3	16.76	51.68	5.91	1172.27	<0.05	668	1.30
CAR	<0.9	<5	0.05	2.25	12.42	12.91	191.80	<0.08	8.36	9.84	<3	13.28	36.89	4.38	888.93	0.43	541	1.50
BER16-A	<0.9	<5	0.11	<0.2	10.84	3.15	172.69	<0.08	5.17	14.75	<3	13.87	37.82	7.23	784.03	0.53	403	6.80
BER16-B	<0.9	3.00	int. Fe	5.42	28.60	5.90	335.00	<0.08	13.80	13.80	<3	34.90	74.10	8.86	1940.00	0.43	1130	9.80
BER16-C	<0.9	<5	<0.2	3.49	14.55	2.03	219.92	<0.08	6.88	16.69	<3	19.29	47.37	5.00	1330.83	0.38	586	30.90
VER	<0.9	3.04	int. Fe	5.78	23.31	6.49	408.45	<0.08	13.07	15.30	<3	33.45	75.30	6.27	1743.24	0.28	882	0.80
RSC16-01_A	<0.9	<5	0.15	2.96	10.51	1.96	255.14	<0.08	4.63	15.42	<3	20.89	39.25	13.65	1598.13	0.25	238	10.30
RSC16-01_B	<0.9	<5	<0.2	2.94	21.41	2.48	150.00	<0.08	5.53	13.11	<3	20.97	34.95	7.96	1134.47	0.50	151	1.80
RSC16-01_C	<0.9	3.52	0.07	4.46	8.79	1.88	394.92	<0.08	5.16	14.18	<3	20.39	36.33	10.30	2145.00	0.20	186	0.70
DRA	<0.9	<5	0.17	9.97	21.13	6.50	486.25	<0.08	8.75	13.75	<3	42.50	95.88	12.93	4487.50	0.88	763	24.10
RSC16-02_A	<0.9	<5	<0.2	3.59	16.67	9.62	405.13	<0.08	10.26	17.31	<3	28.21	64.10	9.15	1500.00	0.28	603	3.00
RSC16-02_B	2.25	6.00	1.62	13.81	68.40	63.00	783.00	1.35	33.00	190.50	4.50	90.45	415.50	317.80	4095.00	4.54	2760	63.60
RSC16-02_C	2.72	7.09	1.19	14.12	54.33	37.80	592.91	1.54	25.98	132.28	3.43	76.54	359.06	164.44	4098.43	2.59	1772	51.20
RSC16-03_A	<0.9	<5	<0.2	3.73	14.52	3.36	276.00	<0.08	7.68	17.40	<3	25.20	61.20	8.48	1452.00	0.30	516	6.10
RSC16-03_B	<0.9	<5	0.39	7.59	20.21	5.32	278.72	<0.08	9.79	28.72	<3	32.13	73.40	18.17	2414.89	0.13	553	11.70
RSC16-03_C	3.21	4.76	1.30	13.92	63.33	71.43	684.52	0.95	32.14	167.86	5.95	86.07	401.19	289.57	3904.76	3.89	2738	62.70
RSC16-04_A	<0.9	<5	0.13	2.83	7.22	1.24	210.90	<0.08	4.74	15.56	<3	13.53	41.73	8.48	744.36	0.26	219	2.50
RSC16-04_B	<0.9	<5	0.27	4.22	14.14	3.20	234.84	<0.08	8.61	16.11	<3	27.05	131.56	8.89	1475.41	0.68	504	11.30
RSC16-04_C	1.23	4.92	0.97	13.68	65.41	60.25	735.25	1.48	30.74	116.80	2.70	83.98	303.69	263.29	4106.56	3.75	2373	60.90
LAI	<0.9	<5	0.53	8.89	37.36	28.16	356.60	<0.08	20.38	12.74	<3	45.28	151.42	23.76	2773.58	2.73	2208	31.50
RSC16-05_A	<0.9	<5	0.08	1.96	10.91	2.41	156.29	<0.08	5.56	14.90	<3	14.37	40.91	8.93	710.14	0.13	283	5.30
RSC16-05_B	<0.9	<5	0.06	<0.2	6.13	1.43	79.17	<0.08	3.65	17.87	<3	8.22	41.74	8.67	455.22	0.53	142	3.30
RSC16-05_C	<0.9	4.05	<0.2	4.75	15.14	4.32	243.24	<0.08	10.95	16.62	<3	24.19	68.92	10.19	1432.43	0.13	635	10.80
RSC16-06_A	<0.9	<5	0.15	5.58	18.21	2.74	422.62	<0.08	9.40	18.81	<3	30.95	69.05	10.32	2035.71	0.13	405	6.10
RSC16-06_B	1.16	<5	0.88	15.23	71.12	55.60	689.22	1.16	32.33	76.29	3.88	82.89	315.52	224.32	3892.24	4.03	2716	74.80
RSC16-06_C	1.05	7.89	1.03	15.80	91.45	65.79	614.47	1.32	35.53	84.21	<3	90.00	386.84	239.63	4223.68	3.48	3618	68.20
RSC16-07_A	<0.9	<5	<0.2	5.83	22.29	3.71	402.86	<0.08	11.71	21.00	<3	34.29	91.43	10.04	1528.57	0.23	671	4.10
RSC16-07_B	1.62	<5	0.81	14.11	69.60	57.52	641.01	1.40	34.53	63.67	2.59	79.64	361.51	161.07	3712.23	4.48	3367	63.70
RSC16-07_C	2.05	5.13	1.23	16.24	83.97	67.44	564.10	1.54	37.18	76.92	3.85	89.23	388.46	269.07	3858.97	5.03	4756	74.60
RSC16-08_A	<0.9	<5	0.06	4.01	13.66	2.32	219.51	<0.08	7.93	20.24	<3	18.29	56.10	17.20	957.32	0.33	182	2.90
RSC16-08_B	<0.9	<5	0.28	13.12	46.74	43.39	731.40	0.62	28.51	34.71	<3	66.32	246.69	62.75	3384.30	3.48	2318	36.70
RSC16-08_C	<0.9	<5	0.37	8.05	34.48	25.40	450.00	<0.08	16.57	22.50	<3	39.92	153.63	32.87	2334.68	1.63	1282	22.30
FLE	0.00	<6	0.35	13.71	51.36	44.49	852.97	<0.08	29.24	20.97	<3	68.64	259.32	80.38	3546.61	6.38	2492	39.20
FLE17-A	0.25	2.83	0.41	10.64	23.50	32.87	512.94	0.67	12.31	32.15	2.70	45.90	142.96	59.64	4405.04	0.80	1103	6.80
FLE17-B	1.64	6.47	4.74	14.00	112.59	121.19	475.09	1.51	31.36	181.02	17.24	83.91	1201.43	321.00	4137.13	4.80	4556	43.70
FLE17-C	0.55	3.81	0.67	9.90	37.63	35.89	487.37	0.77	17.17	27.73	5.37	46.96	196.54	118.41	3249.33	<0.05	3761	48.20

**Table 2:** Geochemical and sedimentological parameters in RSC17 sediment samples (see Table 1 for the signification of abbreviations, and [1] for age-depth model).

ID	Profondeur cm	Age AD	Ag mg/kg	As mg/kg	Cd mg/kg	Co mg/kg	Cr mg/kg	Cu mg/kg	Mn mg/kg	Mo mg/kg	Ni mg/kg	Pb mg/kg	Sn mg/kg	V mg/kg	Zn mg/kg	Hg ng/g	Ti mg/kg	TOC %	S mg/kg	Silts + clays %
*LOD*			*0.15*	*0.5*	*0.09*	*0.15*	*1*	*2*	*0.13*	*0.05*	*0.08*	*0.3*	*0.05*	*0.05*	*0.8*	*0.03*	*0.8*	*0.05*	*50*	
REFERENCE	x	x	0.04	4.58	0.75	14.08	25.96	9.29	474.55	2.29	13.73	12.75	2.05	68.24	207.66	62.33	4344.02	7.90	1243	55.2
RSC16-09_A	2	2017	0.48	2.52	0.08	13.89	32.50	18.54	669.81	0.74	16.90	21.71	3.03	58.92	141.75	19.04	5567.92	0.60	1399	18.1
RSC16-09_B	24	2016	0.31	2.16	0.06	10.61	24.26	9.39	497.01	0.50	13.07	21.81	7.25	45.26	104.79	14.12	4114.29	0.80	1106	14.8
RSC16-09_C	55	2013	0.44	4.68	0.40	13.39	55.49	45.93	1043.19	1.32	33.43	35.79	3.31	72.80	282.59	62.07	4195.06	4.54	3080	56.4
RSC16-09_D	75	2012	0.45	3.70	0.34	12.62	43.09	29.07	682.03	1.16	26.20	30.21	3.54	62.55	226.49	66.75	4026.94	3.79	2628	57.5
RSC17-03_A	133.5	2008	0.29	2.93	0.10	11.54	31.33	19.70	573.74	1.01	19.98	19.61	2.27	50.81	144.17	24.71	4031.66	1.55	1621	24.0
RSC17-03_B	152.5	2007	1.08	3.27	0.19	12.04	30.36	17.07	573.20	0.68	15.66	24.49	5.61	51.82	143.20	46.97	4709.33	1.40	1595	30.4
RSC17-03_C	172.5	2006	1.05	6.11	0.83	15.06	63.95	62.55	638.60	1.56	37.47	48.47	4.48	80.55	356.87	111.04	4216.04	5.09	3838	58.7
RSC17-03_D	193.5	2004	2.39	6.12	1.27	15.23	75.92	80.54	661.78	2.09	39.24	75.08	5.27	87.15	459.69	178.81	4041.88	0.00	4716	67.6
RSC17-04_A	285	1998	0.71	3.83	0.81	11.79	45.01	32.35	587.38	1.10	21.80	33.06	3.19	57.08	222.40	104.85	3844.32	2.81	2245	44.1
RSC17-04_B	314	1996	0.46	2.62	n.a.	11.85	29.78	15.46	559.44	0.90	14.56	20.82	2.33	48.89	143.09	23.39	4201.90	0.73	1469	20.6
RSC17-04_C	334	x	0.18	2.84	0.30	5.98	24.90	19.21	274.31	0.48	11.31	190.11	2.14	27.14	115.83	60.47	1631.08	0.65	2406	7.0
RSC17-04_D	347	1994	3.51	7.05	1.72	16.19	96.78	107.92	531.14	1.93	37.38	92.14	8.37	89.48	504.05	418.21	4154.96	5.34	5994	68.5
RSC17-04_E	367	1993	0.63	5.79	0.46	14.50	67.09	41.05	554.45	1.92	29.08	59.89	3.68	79.22	244.78	145.79	4182.19	3.64	3295	64.3
RSC17-04_F	386.5	x	0.96	4.80	0.89	12.87	53.81	64.24	500.00	1.50	24.61	124.26	14.65	67.24	379.89	188.19	3868.72	4.02	7151	40.3
RSC17-04_G	409	1990	4.46	8.73	8.46	17.00	83.37	123.70	504.98	2.02	40.56	269.43	13.06	103.97	1793.55	348.13	4336.52	5.13	8135	62.9
RSC17-04_H	419.5	1989	2.94	21.20	22.76	13.47	78.74	690.15	386.72	4.64	38.28	262.52	14.65	92.93	4043.16	1375.30	3245.47	17.59	10342	23.4
RSC17-05_A	447.5	1987	0.36	3.56	0.17	8.65	37.28	30.32	401.58	1.03	16.23	72.01	4.87	41.84	373.78	48.96	2600.41	1.29	3236	27.1
RSC17-05_B	467.5	1986	0.28	3.47	0.38	8.14	34.66	31.54	417.05	0.89	15.21	92.03	5.75	39.99	223.99	115.99	2584.72	1.04	3169	18.7
RSC17-05_C	487.5	1985	0.15	2.46	0.39	7.63	28.57	19.82	344.22	0.87	13.00	49.14	4.34	34.08	168.95	56.31	2240.79	0.44	2369	15.5
RSC17-05_D	507.5	1983	0.17	2.15	0.31	8.21	26.11	26.20	408.87	0.70	10.88	67.10	11.59	39.38	134.40	80.57	3301.03	0.94	2323	10.3
RSC17-05_E	534.5	1982	1.95	9.88	2.07	13.38	1448.85	1247.04	476.53	1.31	31.15	252.81	23.94	67.68	1536.04	1606.65	3776.39	9.23	5797	33.6
RSC17-05_F	541.5	1981	2.34	6.47	1.37	12.61	583.41	482.76	475.41	1.04	25.20	171.36	15.56	57.06	779.34	534.26	3650.02	10.40	3842	30.3
RSC17-06_A	599	1977	0.23	4.40	0.51	9.60	97.01	71.65	355.09	0.66	17.59	150.62	5.54	44.49	268.12	143.84	3419.10	14.80	3076	14.5
RSC17-06_B	610	1977	0.44	4.44	0.71	11.56	190.18	152.50	472.13	0.77	19.70	109.60	8.58	55.34	353.29	470.55	3806.79	2.70	2417	28.2
RSC17-06_C	614	1976	0.30	4.41	0.50	11.00	182.00	161.00	447.69	0.64	17.21	81.24	8.07	48.98	293.34	157.93	3499.22	3.50	2399	19.9
RSC17-06_D	637.5	1975	0.49	6.47	0.99	7.98	328.60	197.59	370.65	1.14	16.95	99.25	21.39	48.69	497.24	1700.00	2314.44	19.16	4922	n.a.
RSC17-06_E	641.5	1974	0.34	4.06	0.53	11.08	163.53	147.51	447.43	0.71	18.28	82.33	7.72	50.88	293.86	185.69	3493.00	3.90	2376	23.7
RSC17-06_F	668.5	1973	0.92	5.38	1.19	9.49	589.03	466.33	395.64	1.42	19.46	132.47	22.02	47.00	717.67	545.77	2851.07	7.50	5097	24.4
RSC17-06_G	700.5	x	0.14	2.56	0.48	6.26	27.47	39.63	345.17	0.62	12.94	50.78	9.46	31.29	141.22	45.28	1590.17	0.40	2488	5.3

This article also includes the calculation of three pollution indexes: enrichment factors (EF), geoaccumulation indexes (Igeo), and the metallic pollution index (MPI) for the two datasets displayed in Table 1, Table 2, respectively. Contamination categories for EF and Igeo are listed in Table 3. Results and interpretations of EF, Igeo, and MPI are presented in two Excel sheets in supplementary data.

**Table 3:** Contamination categories based on Enrichment factors (EF) and Geoaccumulation index (Igeo).

Enrichment factors (EF)^a^			Geoaccumulation index (Igeo)^b^		
Level	Value	Enrichment	Class	Value	Contamination
I	<1	none	0	<0	none
II	1–3	minor	1	0–1	none to moderate
III	3–5	moderate	2	1–2	moderate
IV	5–10	moderately severe	3	2–3	moderate to strong
V	10–25	severe	4	3–4	strong
VI	25–50	very severe	5	4–5	strong to extreme
VII	>50	extremely severe	6	>5	extreme

a According to Chen et al. (2007).

b According to Muller (1981).

## 2 Experimental design, materials, and methods

### 2.1 CHNS analyzer

Total Carbon (TC) and Total Organic Carbon (TOC) contents were determined using a CHNS analyzer TruSpec® Leco 932 (catalytic combustion method and infrared detection), with a Limit of Detection (LOD) of 0.05% and a Limit of Quantification (LOQ) of 0.17%, respectively. The sample set was first dried during 24h at 50 °C and then analyzed for the assessment of TC content. The same set was used for TOC measurements by using silver capsules. They were placed on a plastic plate with small numbered wells. The samples were then moistened with about 20μL of ELGA water which allowed acidification. The plate was then placed in a sealed glass desiccator in the presence of a small beaker containing about 25mL of concentrated HCl. The samples were exposed to HCl steam for 4 hours at room temperature. They were then removed and placed in the oven for 1 hour at 50 °C to remove HCl and water residues. The capsules were then closed and placed in the CHNS analyzer without reweighing. Analyses were performed in duplicates using PACS-2 (Marine sediment) and OAS as standard reference materials for control. For additional information about certified reference values, the reader is referred to supplementary data.

### 2.2 Grain-size analyses

Grain-size analyses were performed by sieving the coarse fraction using apertures of 16, 11.3, 8, 5.6, 4, 2.8, 2, 1.4, and 1 mm. Laser diffraction was performed without pretreatment to characterize the fraction under 2 mm in duplicate or triplicate using a Horiba® LA-950 Laser Particle Size Analyzer. Data were then combined and interpreted using the Folk and Ward method [2] in the GRADISTAT Excel spreadsheet [3] to extract parameters such as silt and clay contents and d50.

### 2.3 ICP-AES

Total acid attacks were performed on ca. 0.1 g of crushed sediment by mixing 4 ml of HNO_3_ with 1.6 ml of HClO_4_, and 2 ml of HF in Teflon tubes completed to 15 ml with ultrapure water. The quantification of major elements and trace metals, except for mercury, have been performed using an Inductively Coupled Plasma Atomic Emission Spectrometry (ICP-AES) Varian X® with multi-elements solutions, reference materials and sample replicates. LSKD-2, LSKD-4 (Lake sediments) and Buffalo RM8704 (River sediment) were used as certified reference materials (SI).

### 2.4 AAS

Mercury (Hg) content was analyzed on ca. 50 mg of dried powders following thermal decomposition, amalgamation, and Atomic Absorption Spectroscopy (AAS) analyses using a DMA-80 with an instrumental LOD of 0.005 ng/g of sediment. Different certified control masses of known concentrations were analyzed to make a calibration curve ranging from 1 to 25 ng of Hg. For each analysis, the sample is heated to 200 °C for 1min, then the temperature increases for 1min30s to reach 650 °C. This temperature is maintained for another 1min30s. During this time, the Hg steam is captured in the "amalgamator" containing gold, which captures Hg. After 1min30sec. At 650 °C, the "amalgamator" is heated to 900 °C for 12s, which releases the Hg that goes into the detection cell. Hg is then detected by AAS at 253.65nm. This method allowed to determine a mean LOD of 0.03 ng/g of sediment for the whole dataset, which varies according to the mass and the Hg concentration of each sample.

### 2.5 Pollution indexes

The assessment of pollution was made by calculating Enrichments Factors (EF, equation 1) [4], [5], [6] and Geoaccumulation Indexes (Igeo, equation 2) [7], [8]. EF and Igeo are both seven classes indexes used to assess a pollution by a single metal (Table 3).

(1) EF (X) = $\frac{\left(X/\left(Ti\right)sample\right)}{\left(X/\left(Ti\right)ref\right)}$ where X and Ti represent the metal and titanium concentrations, respectively, in sample or reference sample in mg kg^−1^.
(2) Igeo (X) = $log2\times \frac{Xsample}{1.5\;\times \;Xref}$ where X represents the metal concentration in sample or reference sample in mg kg^−1^.

**Fig. 1:**
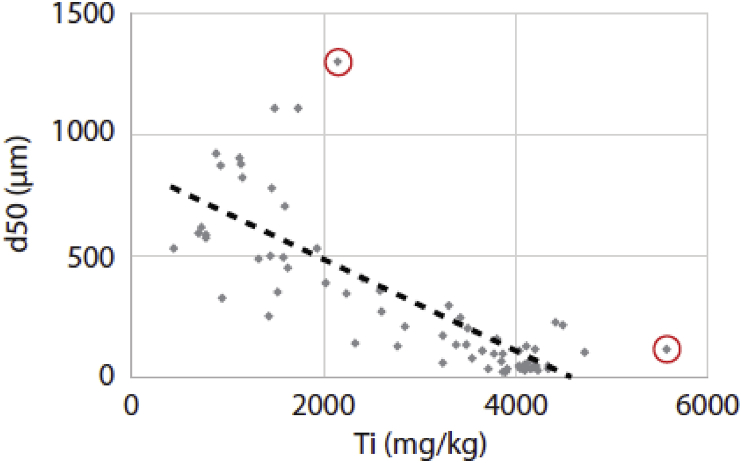
Linear regression between Ti concentration and the d50 value (grain-size analysis) showing a negative and significant relationship (y = −0.1883x + 863.54). Red circles refer to outliers. See text for details.

The calculation of EFs requires a reference sample for background geochemical values and a conservative element to normalize geochemical data that can be affected by grain-size effect. The reference sample was provided by sampling a deep layer in a core (LSC17) collected in the lake feeding the Saint-Charles 30 km upstream [1]. According to the age-depth model presented in Tremblay et al. [9], the layer sampled at 85–86 cm depth in core LSC17 predates the European settlement in Canada and was thus targeted to evaluate natural background concentrations for metals [1]. The affinity of Ti for fine sediments was first suggested from Itrax® data [1], and then confirmed when plotting Ti inferred from ICP-AES analyses versus grain-size. Fig. 1 shows the relationship between Ti and d50 is negative (y = -0.1883x + 863.54; r = 0.79) and significant (p < 10^−4^). This relationship is even stronger (r = 0.83) when two outliers are removed from the dataset.

We inferred the extent of polymetallic contamination for each sample by calculating Metallic Pollution Index (MPI, equation 3) [10]. MPI values > 1 indicate pollution whereas MPI values < 1 indicate no pollution.

(3) MPI = ${\left(\frac{M1sample}{M1ref}\times \frac{M2sample}{M2ref}\times \frac{M3sample}{M3ref}\times \ldots \;\times \frac{Mnsample}{Mnref}\right)}^{1/n}$ where M represents the metal concentration whereas n indicates the number of metals considered.
