# Supplementary material for: Dataset for the assessment of metallic pollution in the Saint-Charles River sediments (Québec City, QC, Canada)
Source: Data Brief. 2019 Jul 15;26:104256. doi: 10.1016/j.dib.2019.104256 (PMC6731351; doi:10.1016/j.dib.2019.104256)
Supplement: Supplementary file 1 [file mmc1.pdf]

National Institute of Standards & Technology

Report of Investigation

Reference Material 8704

Buffalo River Sediment

This Reference Material (RM) is intended primarily for use in the analysis of sediments, soils, or materials of a similar matrix. A unit of RM 8704 consists of 50 g of freeze-dried, radiation-sterilized, homogenized, river sediment.

Reference Mass Fraction Values: Reference values for the mass fractions of 25 elements are given in Table 1. Reference values are noncertified values that are the best estimate of the true value; however, the values do not meet the NIST criteria for certification and are provided with associated uncertainties that may not include all sources of uncertainty [1].

Information Mass Fraction Value: An information value for arsenic is provided in Table 2. An information value is a noncertified value for which there is insufficient information to assign an uncertainty [1]. An information value cannot be used to establish metrological traceability.

Expiration of Values Assignment: **RM 8704** is valid, within the measurement uncertainties specified, until **01 December 2027**, provided the RM is handled and stored in accordance with instructions given in this Report of Investigation (see “Instructions for Use”). This report is nullified if the RM is damaged, contaminated, or otherwise modified.

Maintenance of Reference Values: NIST will monitor this RM over the period of its validity. If substantive technical changes occur that affect the reference values, NIST will notify the purchaser. Registration (see attached sheet or register online) will facilitate notification.

Overall direction and coordination of the analyses were provided by G.C. Turk formerly of NIST.

Spectrometric measurements were performed by A.P. Lindstrom of the NIST Materials Measurement Science Division and G.C. Turk, L.J. Wood, and L.L. Yu of the NIST Chemical Sciences Division. Instrumental Neutron Activation Analysis was performed by R.R. Greenberg of the NIST Chemical Sciences Division.

Statistical consultation was provided by W.F. Guthrie of the NIST Statistical Engineering Division.

Support aspects involved in the issuance of this RM were coordinated through the NIST Office of Reference Materials.

Carlos A. Gonzalez, Chief
Chemical Sciences Division

Steven J. Choquette, Director
Office of Reference Materials

Gaithersburg, MD 20899
Report Issue Date: 24 July 2018
Report Revision History on Last Page

SOURCE, PREPARATION, AND ANALYSIS[(1)]

Source and Preparation of Material: The river sediment for SRM 2704 *Buffalo River Sediment* and RM 8704 *Buffalo River Sediment* was collected from the Buffalo River in the area of the Ohio Street Bridge (Buffalo, NY). The U.S. Army Corps of Engineers, under contract to NIST, collected and screened approximately 908 kg of river sediment and placed it in six 55-gallon, Teflon®-lined drums. The drums were loaded onto a refrigerated truck and transported to the Technimed Corporation (Fort Lauderdale, FL) for freeze-drying of the contents. The freeze-dried sediment was shipped to a laboratory contracted by NIST where it was screened and passed through a 150 µm (100 mesh) sieve and retained on a 38 µm (400 mesh) sieve. The SRM 2704 portion of the freeze-dried sediment was sieved and blended at NIST and subsequently radiation-sterilized, and bottled into 50 g units. The RM 8704 portion of the freeze-dried and sieved sediment was retained for approximately 6 years before being blended, radiation-sterilized, divided by a spinning riffler, and bottled into 50 g units.

Spectrometric Comparison to SRM 2704: Samples weighing approximately 0.25 g were taken from each of 8 bottles of RM 8704 and 8 bottles of SRM 2704. The samples were put into solution using a lithium metaborate fusion procedure. The solutions of the two materials were spectrometrically compared using both inductively coupled plasma mass spectrometry (ICPMS) and inductively coupled plasma optical emission spectrometry (ICPOES). Mass fraction values for 18 elements in RM 8704 were calculated from the relative signal intensities (optical and/or mass spectrometric) of RM 8704 versus SRM 2704 and the certified mass fractions of SRM 2704. ICPMS measurements were made for arsenic, barium, calcium, cadmium, cobalt, chromium, iron, magnesium, nickel, lead, titanium, uranium, vanadium, and zinc. For some elements ICPMS signals were measured at more than one mass, and the equally-weighted mean value was used as the ICPMS value. ICPOES measurements were made for aluminum, barium, calcium, cobalt, chromium, iron, potassium, magnesium, manganese, sodium, lead, titanium, uranium, vanadium, and zinc. For the elements for which both ICPMS and ICPOES measurements were made, the equally-weighted mean value was used.

Combustion Analysis: Carbon was determined in 8 bottles of RM 8704 and 8 bottles of SRM 2704 at LECO Corporation, Inc. using combustion analysis with infrared detection. In a manner analogous to the spectrometric comparison, the mass fraction of carbon was calculated from the relative carbon signals of RM 8704 versus SRM 2704 and the certified mass fraction of carbon in SRM 2704. The sample size was 0.25 g.

Instrumental Neutron Activation Analysis (INAA): Samples weighing approximately 0.25 g were taken from each of 8 bottles of RM 8704 and 2 bottles of SRM 2704. Using standards prepared from dried filter papers onto which known amounts of the analytes of interest had been deposited, cerium, cobalt, chromium, cesium, europium, iron, hafnium, antimony, scandium, and thorium, were determined by INAA. SRM 2704 was analyzed as a quality control sample.

INSTRUCTIONS FOR USE

Use: A minimum sample weight of 0.25 g (dry weight, see "Instructions for Drying") should be used for analytical determinations relating to the reference values on this report of investigation.

Sample preparation procedures should be designed to effect complete dissolution. If volatile elements (i.e., mercury, arsenic, selenium) are to be determined, precautions should be taken in the dissolution of RM 8704 to avoid volatilization losses.

Instructions for Drying: When nonvolatile elements are to be determined, samples should be dried for 2 hours at 110 °C. Volatile elements (i.e., mercury, arsenic, selenium) should be determined on samples as received; separate samples should be dried as previously described to obtain a correction factor for moisture. Correction for moisture is to be made to the data for volatile elements before comparing to the reference values. This procedure, which was used for the determination of the volatile elements, ensures that these elements are not lost during drying. The approximate mass loss on drying has been found to be 0.8 %.

[(1)]Certain commercial equipment, instruments or materials are identified in this report to adequately specify the experimental procedure. Such identification does not imply recommendation or endorsement by the National Institute of Standards and Technology, nor does it imply that the materials or equipment identified are necessarily the best available for the purpose.

Table 1. Reference Mass Fraction Values for Elements for RM 8704[(a)]

	Mass Fraction (%)		Mass Fraction (%)
Aluminum (Al)	6.10 ± 0.18	Magnesium (Mg)	1.200 ± 0.018
Calcium (Ca)	2.641 ± 0.083	Potassium (K)	2.001 ± 0.041
Carbon (C)	3.351 ± 0.017	Sodium (Na)	0.553 ± 0.015
Iron (Fe)	3.97 ± 0.10	Titanium (Ti)	0.457 ± 0.020

	Mass Fraction (mg/kg)		Mass Fraction (mg/kg)
Antimony (Sb)	3.07 ± 0.32	Lead (Pb)	150 ± 17
Barium (Ba)	413 ± 13	Manganese (Mn)	544 ± 21
Cadmium (Cd)	2.94 ± 0.29	Nickel (Ni)	42.9 ± 3.7
Cerium (Ce)	66.5 ± 2	Scandium (Sc)	11.26 ± 0.19
Cesium (Cs)	5.83 ± 0.12	Thorium (Th)	9.07 ± 0.16
Chromium (Cr)	121.9 ± 3.8	Uranium (U)	3.09 ± 0.13
Cobalt (Co)	13.57 ± 0.43	Vanadium (V)	94.6 ± 4.0
Europium (Eu)	1.31 ± 0.038	Zinc (Zn)	408 ± 15
Hafnium (Hf)	8.4 ± 1.5		

[(a)] The measurand is the mass fraction of each vitamin listed in Table 1 as determined by the method indicated above. Metrological traceability is to mass fraction (expressed as milligrams per kilogram or percent), as realized by the methods used.

Reference Value Uncertainties: The uncertainty in the reference values for aluminum, calcium, potassium, and sodium, derived from spectrometric comparison to SRM 2704 using ICPOES, is expressed as an expanded uncertainty, U, at the 95 % level of confidence, and is calculated according to the ISO/JCGM Guides [2]. The expanded uncertainty is calculated as $U = ku_c$, where u_c is intended to represent, at the level of one standard deviation, the combined effects of the ICPOES measurement uncertainty and the uncertainty of the SRM 2704 certified value. The uncertainty for the SRM 2704 certified value has been recalculated from the original data according to ISO/JCGM measurement uncertainty guidelines. The coverage factor, k, was obtained from the Student's t-distribution corresponding to the calculated effective degrees of freedom of u_c using a level of confidence of 95 %.

The uncertainty in the reference values for cadmium and nickel, derived from spectrometric comparison to SRM 2704 using ICPMS, is expressed as an expanded uncertainty, U, at the 95 % level of confidence, and is calculated according to the ISO/JCGM Guides [2]. The expanded uncertainty is calculated as $U = ku_c$, where u_c is intended to represent, at the level of one standard deviation, the combined effects of the ICPMS measurement uncertainty and the uncertainty of the certified value of SRM 2704. The uncertainty for the certified value of SRM 2704 has been recalculated from the original data according to ISO/JCGM measurement uncertainty guidelines. The coverage factor, k, was obtained from the Student's t-distribution corresponding to the calculated effective degrees of freedom of u_c using a level of confidence of 95 %.

The uncertainty in the reference values for magnesium, titanium, barium, manganese, lead, uranium, vanadium, and zinc, derived from spectrometric comparison to SRM 2704 using the equally weighted mean of ICPMS and ICPOES measurements, is expressed as an expanded uncertainty, U, at the 95 % level of confidence, and is calculated according to the ISO/JCGM Guides [2]. The expanded uncertainty is calculated as $U = ku_c$, where u_c is intended to represent, at the level of one standard deviation, the combined effects of the ICPOES measurement uncertainty, the ICPMS measurement uncertainty, and the uncertainty of the SRM 2704 certified value. The uncertainty for the SRM 2704 certified value has been recalculated from the original data according to ISO/JCGM measurement uncertainty guidelines. The coverage factor, k, was obtained from the Student's t-distribution corresponding to the calculated effective degrees of freedom of u_c using a level of confidence of 95 %.

The uncertainty in the reference values for iron, cobalt, and chromium, derived from the equally weighed mean of a spectrometric comparison to SRM 2704 (using both ICPMS and ICPOES) and an independent INAA analysis, is expressed as an expanded uncertainty, U, at the 95 % level of confidence, and is calculated according to the ISO/JCGM and NIST Guides [2]. The expanded uncertainty is calculated as $U = ku_c$, where u_c is intended to represent, at the level of one standard deviation, the combined effects of the uncertainty in the spectrometric comparison (which includes ICPMS measurement uncertainty, ICPOES measurement uncertainty, and the uncertainty of the SRM 2704 certified value), the uncertainty of the INAA measurement, and the uncertainty of corrections for method biases [3]. The uncertainty for the SRM 2704 certified value has been recalculated from the original data according to ISO/JCGM

measurement uncertainty guidelines. The coverage factor, k, was obtained from the Student's t-distribution corresponding to the calculated effective degrees of freedom of u_c using a level of confidence of 95 %.

The uncertainty in the reference values of cerium, cesium, europium, hafnium, antimony, scandium, and thorium, derived from INAA analysis alone, is expressed as an expanded uncertainty, U, at the 95 % level of confidence, and is calculated according to the ISO/JCGM Guides [2]. The expanded uncertainty is calculated as $U = ku_c$, where u_c is intended to represent, at the level of one standard deviation, the uncertainty of the INAA measurements. The coverage factor, k, was obtained from the Student's t-distribution corresponding to the calculated effective degrees of freedom of u_c using a level of confidence of 95 %.

The uncertainty in the reference value of carbon, derived from comparison to SRM 2704 using combustion analysis, is expressed as an expanded uncertainty, U, at the 95 % level of confidence, and is calculated according to the ISO/JCGMGuides [2]. The expanded uncertainty is calculated as $U = ku_c$, where u_c is intended to represent, at the level of one standard deviation, the combined effects of the combustion analysis measurement uncertainty and the uncertainty of the SRM 2704 certified value. The uncertainty for the SRM 2704 certified value has been recalculated from the original data according to ISO/JCGM measurement uncertainty guidelines. The coverage factor, k, was obtained from the Student's t-distribution corresponding to the calculated effective degrees of freedom of u_c using a level of confidence of 95 %.

Table 2. Information Mass Fraction Value for Arsenic for RM 8704

Element	Mass Fraction (mg/kg)
Arsenic	17

Information Value for Arsenic: This information value was determined by spectrometric comparison to SRM 2704 using ICPMS after adjusting the certified value of arsenic in SRM 2704 downward by 6 %. The adjustment is based on measurements made at NIST since 1996, that indicate the certified value for SRM 2704 is no longer valid due to a loss of approximately 6 % of the arsenic from SRM 2704. The arsenic value in RM 8704 is given for information only because there is insufficient information to assign an uncertainty.

REFERENCES

[1] May, W.; Parris, R.; Beck II, C.; Fassett, J.; Greenberg, R.; Guenther, F.; Kramer, G.; Wise, S.; Gills, T.; Colbert, J.; Gettings, R.; MacDonald, B.; *Definitions of Terms and Modes Used at NIST for Value-Assignment of Reference Materials for Chemical Measurements*; NIST Special Publication 260-136; U.S. Government Printing Office: Washington, DC (2000); available at: https://www.nist.gov/srm/publications.cfm (accessed July 2018).

[2] JCGM 101:2008; *Evaluation of Measurement Data — Supplement 1 to the "Guide to the Expression of Uncertainty in Measurement" — Propagation of Distributions using a Monte Carlo Method*; Joint Committee for Guides in Metrology (JCGM) (2008) available at https://www.bipm.org/utils/common/documents/jcgm/JCGM_101_2008_E.pdf (accessed July 2018).

[3] Levenson, M.S.; Banks, D.L.; Eberhardt, K.R.; Gill, L.M.; Guthrie, W.F.; Liu, H.-K.; Vangel, M.G.; Yen, J.H.; Zhang, N.F.; *An Approach to Combining Results From Multiple Methods Motivated by the ISO GUM*; J. Res. Natl. Inst. Stand. Technol., Vol. 105; p. 571 (2000).

Report Revision History: 24 July 2018 (Change of expiration date; editorial changes); 30 January 2013 (Removed copper and thallium from list of elements analyzed by ICPMS); 08 January 2008 (Update of expiration date and editorial changes); 03 March 2000 (Original report date).

Users of this RM should ensure that the Report of Investigation in their possession is current. This can be accomplished by contacting the SRM Program: telephone (301) 975-2200; fax (301) 948-3730; e-mail srminfo@nist.gov; or via the Internet at https://www.nist.gov/srm.

LKSD-1 à LKSD-4

Sédiments lacustres

Les matériaux LKSD-1 à LKSD-4 sont des échantillons lacustres caractéristiques prélevés en divers endroits du Bouclier canadien. On a fait en sorte que ces échantillons présentent une gamme de concentrations pour un nombre important d'éléments. La Commission géologique du Canada a effectué les prélèvements à l'aide de divers dispositifs de dragage et d'échantillonnage à la fois en hiver et en été, normalement au centre du lac. Les échantillons ont été préparés, mélangés et embouteillés à CANMET. Les renseignements concernant chaque échantillon sont donnés ci-dessous. Les repères cartographiques sont ceux du Système national de référence cartographique (SNRC).

LKSD-1 : Cet échantillon est un mélange de lots provenant du lac Joe (31F) et du lac Brady (31M) en Ontario.

LKSD-2 : Cet échantillon est un mélange d'un lot provenant du lac Calabogie (31F) en Ontario et d'un composite de 86K et 86L dans les Territoires du Nord-Ouest. Les composites ont été obtenus en mélangeant des fractions non utilisées d'échantillons recueillis lors de levés régionaux dans les feuilles correspondantes du SNRC.

LKSD-3 : Cet échantillon est un mélange d'un lot provenant du lac Calabogie (31F) en Ontario, d'un composite de 64L et 64M au Manitoba et d'un composite de 31M, 31N, 32C, 32D, 41P et 42A en Ontario.

LKSD-4 : Cet échantillon est un composite du lac Big Gull (31C) en Ontario et des lacs Key et Sea Horse (74H) en Saskatchewan.

Trente-cinq laboratoires ont fourni des résultats d'analyses et des valeurs recommandées provisoires sont données pour 65 éléments. En plus des valeurs "totales", les échantillons ont également été caractérisés pour des valeurs liées à des types particuliers d'extraction partielle, lorsque l'échantillon n'est pas complètement dissous; c'est le cas en particulier des silicates. Les géochimistes et les scientifiques de l'environnement effectuent souvent ce type d'analyse et ces échantillons de référence devraient leur être utiles.

Un rapport fournissant des explications complètes sur ces sédiments lacustres de référence sera expédié avec chaque commande d'échantillons LKSD-1 à LKSD-4 qui ne sont disponibles qu'en série de quatre.

Valeurs provisoires pour les éléments majeurs et mineurs sous forme d'oxydes, exprimées en pourcentage

Constituant	LKSD-1	LKSD-2	LKSD-3	LKSD-4
SiO_2	40.1	58.9	58.5	41.6
Al_2O_3	7.8	12.3	12.5	5.9
Fe_2O_3	4.1	6.2	5.7	4.1
MgO	1.7	1.7	2.0	0.9

CaO	10.8	2.2	2.3	1.8
Na_2O	2.0	1.9	2.3	0.7
K_2O	1.1	2.6	2.2	0.8
MnO	0.1	0.3	0.2	0.1
TiO_2	0.5	0.6	0.5	0.4
P_2O_5	0.2	0.3	0.2	0.3
LOI (1000°C)	29.9	13.6	13.4	43.6
SO_4*	1.6	-	-	-
Sum	99.9	100.6	99.8	100.2

* un laboratoire seulement

Valeurs provisoires pour les éléments "totaux"
g/g (sauf indication contraire)

Constituant	LKSD-1	LKSD-2	LKSD-3	LKSD-4
Ag	0.6	0.8	2.7	<0.5
As	40	11	27	16
Au (ng/g)	5	3	3	2
B	49	65	25	22
Ba	430	780	680	330
Be	1.1	2.5	1.9	1.0
Br	11	18	16	49
C (%)	12.3	4.5	4.5	17.7
Ce	27	108	90	48
Co	11	17	30	11
Cr	31	57	87	33
Cs	1.5	3.0	2.3	1.7
Cu	44	37	35	31
Dy	3.4	7.3	4.9	3.7
Eu	0.9	1.9	1.5	1.1
F	300	590	490	260
Fe (%)	2.8	4.3	4.0	2.8
H_2O- (%)	2.92	2.23	2.07	6.55
Hf	3.6	7.0	4.8	2.8
La	16	68	52	26
Li	7	20	25	12
LOI(500°C)(%)	23.5	12.3	11.8	40.8

Lu	0.4	0.6	0.4	0.5
Mn	700	2020	1440	500
Mo	10	<5	<5	<5
Nb	7	8	8	9
Nd	16	58	44	25
Ni	16	26	47	31
Pb	82	44	29	91
Rb	24	85	78	28
S (%)	1.57	0.14	0.14	0.99
Sb	1.2	1.1	1.3	1.7
Sc	9	13	13	7
Sm	4	11	8	5
Sn	16	5	3	5
Sr	250	220	240	110
Ta	0.3	0.8	0.7	0.4
Tb	0.6	1.4	1.0	1.2
Th	2.2	13.4	11.4	5.1
Ti	3010	3460	3330	2270
U	9.7	7.6	4.6	31.0
V	50	77	82	49
W	<4	<4	<4	<4
Y	19	44	30	23
Yb	2.0	4.0	2.7	2.0
Zn	331	209	152	194
Zr	134	254	178	105

Valeurs provisoires pour les éléments partiellement extraits
HNO_3 concentré - HCl concentré
g/g (sauf indication contraire)

Constituant	LKSD-1	LKSD-2	LKSD-3	LKSD-4
Ag	0.6	0.8	2.4	0.2
As	30	9	23	12
Cd	1.2	0.8	0.6	1.9
Co	9	17	30	11
Cr	12	29	51	21
Cu	44	36	34	30
Fe (%)	1.8	3.5	3.5	2.7

Hg (ng/g)	110	160	290	190
Mn	460	1840	1220	430
Mo	12	2	2	2
Ni	11	23	44	32
Pb	84	40	26	93
Sb	1.2	1.2	1.4	1.5
V	27	48	55	32
Zn	337	200	139	189

Valeurs provisoires pour les éléments partiellement extraits
HNO_3 dilué - HCl dilué
g/g (sauf indication contraire)

Constituant	LKSD-1	LKSD-2	LKSD-3	LKSD-4
Ag	0.6	0.8	2.8	0.2
Cd	1.2	0.6	0.4	1.9
Co	8	16	30	9
Cu	44	36	34	31
Fe (%)	1.8	3.7	3.6	2.6
Mn	410	1840	1300	420
Ni	12	23	46	31
Pb	83	34	21	91
Zn	335	205	151	195

PCMR - CANMET (NRCan)
555, rue Booth
Ottawa, (Ontario) Canada
K1A 0G1

Téléphone : (613) 995-4738
Télécopieur : (613) 943-0573
Courriel: pcmr@nrcan.gc.ca
